# Compound asset sharing initiatives between pharmaceutical companies, funding bodies, and academia: Learnings and successes

**DOI:** 10.1002/prp2.510

**Published:** 2019-07-30

**Authors:** Ann G. Hayes, David J. Nutt

**Affiliations:** ^1^ The Ann Hayes Consultancy Buntingford Hertfordshire UK; ^2^ Imperial College London Hammersmith Hospital London UK

## Abstract

Over recent years, there have been several initiatives to gain access to compounds which have been deprioritized by pharmaceutical companies, but which have a data package allowing them to be used in human experimental medicine studies. Such compounds provide an invaluable resource for probing human biology and disease pathology, for improving translational capabilities, and ultimately for repurposing to new therapeutic indications. The authors have been involved with the setting up of the Medicine's Chest initiative, which aimed to access compounds for the use in clinical studies of the central nervous system. Other initiatives include those set up by AstraZeneca, the Medical Research Council in the UK and the National Center for Advancing Translational Sciences in the US. The purpose of this editorial is to provide an update on progress with some of these initiatives, and to identify some learnings for future endeavours.

Despite some notable successes in the introduction of innovative new medicines, for example, in the areas of oncology, multiple sclerosis, and hepatitis C, attrition rates for pharmaceutical development remain high, particularly for psychiatric diseases like depression and neurological diseases like Alzheimer's. These high attrition rates reflect a number of issues, particularly a lack of understanding of human physiology and human disease etiology and pathology and this has led to a lack of predictivity of animal models and a failure of translation from preclinical to clinical studies. This has been particularly acute for CNS diseases. Historically, many drugs for treating CNS diseases were discovered by serendipity, during their use in the clinic for another indication. It has been recognized that such “repurposing” of drugs remains a useful strategy in all therapeutic areas and there are some excellent examples, like the repurposing of thalidomide for cancer treatment.

Use of compounds for both increasing understanding of human biology and for repurposing requires the availability of suitable compounds for use in clinical studies, drug substance/product for administration and an accompanying regulatory package allowing its use in humans. This regulatory package would include the required pharmacological, toxicological, and pharmacokinetic information to allow the drug to be dosed in humans. There have been a number of initiatives to access such compounds for human use, where they have been through preclinical and clinical development within a pharmaceutical company, but have been terminated before commercialization for non‐safety reasons, like lack of efficacy, or for strategic reasons. The purpose of this editorial is to provide an update on progress with some of these initiatives, and to identify some learnings for future endeavours.

The authors were instrumental in setting up the Medicine's chest initiative (https://www.ecnp.eu/research-innovation/ECNP-medicines-chest), funded by the European College of Neuropsychopharmacology (ECNP).^1^ The Medicine's chest concentrated on accessing compounds which had been developed for diseases of the Central Nervous System (CNS). This emerged to support the interests of ECNP, but more importantly reflected the difficulties in translation for CNS diseases and the high attrition rates, and hence the need for different strategies for drug development. The main objective for the Medicine's chest has been to gain access to tool compounds, selective for particular brain pathways or receptors, for use by academics and small companies in pharmacological experiments in human beings, to gain information about molecular targets, CNS pathways, and human neurobiology/pathology. Although the primary aim was not repurposing, it was always hoped that an increase in understanding would ultimately help in the search for innovative new drugs. This requires potent and selective compounds that access the CNS and have the required regulatory package for use in humans.

There are a number of compounds in the Medicine's chest, including a selective dopamine D1 receptor antagonist, ADX10061, a selective 5HT2a receptor antagonist, volinanserin, and an alpha2 adrenoceptor receptor agonist, idazoxan. Although there has been considerable interest in accessing the compounds from the academic community, it has been hard to progress studies because there is no availability of drug substance for some compounds. We have access to the required regulatory documentation to allow a resynthesis and formulation of many of the compounds to standards of “Good Manufacturing Practice” (GMP), and the original plan had been to encourage researchers to apply for money for resynthesis as part of their grant applications for doing the clinical studies. However, we felt that this was a “road‐block” and therefore applied to the Wellcome Trust for a Biomedical resources Grant for the production of one of the compounds, the D1 antagonist ADX10061. This grant was awarded in September 2018 and synthesis of the compound is underway.^2^ The grant is allowing the resynthesis of about 1 kg of drug substance by a Contract Manufacturing Organisation and then it's formulation into capsules, with matching placebo capsules. Drug substance and product will then be stored and distributed by the manufacturer. Initially, the compound will be used in studies involving a collaboration between investigators at Imperial College London, Cambridge University and Oxford University and will cover a range of topics from understanding of cognition and attention, prefrontal cortex, and basal ganglia function, through to addiction and depression. But it is planned that sufficient drug (and matching placebo) will be made to allow other researchers to access it for further human studies. Importantly, the originating company, Addex, has provided both expertise and regulatory documentation which is proving very helpful. In return, the clinical studies using ADX10061 may deliver important new information about the role of D1 receptors in the brain and potentially new intellectual property which could be licensed by Addex, if in an area of interest to them. Access to such intellectual property is being agreed within a contract which is being drawn up between the participating universities and Addex.

Other initiatives for gaining access to shelved compounds from Pharma include:

- Repurposing initiatives from AstraZeneca, in collaboration with various grant awarding bodies, including the Medical Research Council (MRC) in the UK, the “Mechanisms for Human Diseases Initiative,”^3^ the National Institutes of Health (NIH) in the US, and The Innovative Medicines Initiative (IMI) in the European Union.
- An initiative of the MRC to investigate human mechanisms of disease, which could potentially lead to new therapies; the MRC has launched a number of calls tied to their compound list, the MRC/AstraZeneca call described above, the MRC‐Industry Asset Sharing Initiative, and Experimental Medicine Challenge grants.
- An initiative to test process improvements in translational science called the “New Therapeutic Uses: NIH‐Industry Partnerships Initiative” launched by the National Center for Advancing Translational Sciences (NCATS) within NIH.^4^

All of these asset sharing initiatives seem to have similar objectives, that is, the use of pharmacological studies in humans to better understand human biology and disease, to improve translation from preclinical to clinical studies, and ultimately to help discover new medicines. Some of the initiatives provide assets for both preclinical and clinical studies, but the main aim was to collect clinical data. Apart from the Medicine's chest, they are all disease agnostic and cover all therapy areas.

A key barrier for setting up industry/academia/government collaborations has often been getting suitable legal agreements signed off in a reasonable time frame. This has been largely overcome by the MRC and NCATS by the development of template contracts that have been pre‐agreed with participating Pharma companies; the NCATS templates are publicly available on the NCATS website (https://ncats.nih.gov/ntu/assets/agreements). The Medicine's chest publishes a number of template agreements on the ECNP website (available to members only), and our expert consultants will help broker these agreements. NCATS report that that the use of these templates has led to a decrease in the time taken to establish collaborations from the standard 9‐12 months to around 3‐4 months, which is a considerable gain.

Unlike the Medicine's chest, the other initiatives generally involve the Pharma companies providing drug product as well as regulatory documentation. This is a distinct advantage, although it does limit the compounds available for asset sharing initiatives, as even if companies have API (Active Pharmaceutical Ingredient) available, it may be out of the specification required for clinical use or need formulating into drug product, which can be very expensive. If such initiatives are to gain real traction, it may be necessary for funding bodies to think about providing grant monies for these activities. Pharma is unlikely to be willing to provide significant budget, as funds will be tied up with competing priorities, although AstraZeneca have spent a considerable amount of money on resynthesis and drug product supply.

All the initiatives report that a key aspect of success for studies on shelved drugs is the expertise provided by the donating Pharma company; this is especially meritorious given that the companies’ priorities will have shifted elsewhere and company personnel will have to do this in addition to their “day‐job.” We have mentioned above the example of Addex, which is a small company, but is providing input to facilitate the resynthesis of ADX10061. AstraZeneca has provided advice and expertise on their drugs, as well as budget. Other expertise provided by companies includes information on human safety studies, toxicity testing, and drug dosing, which is critical for success and often underappreciated by academics.

Within the Medicine's chest, we have negotiated access to compounds for human use, but investigators need to raise grant monies from other sources to actually carry out the clinical research studies. Several grant awarding bodies have indicated their willingness to provide grants, for example the Stanley Foundation, the MRC (UK only), and the Wellcome Trust. The NCATS and MRC initiatives provide access to grant funds as well as access to compounds, which is a considerable advantage.

Some of these initiatives have been running for several years now, and it is encouraging to report that this has resulted in considerable activity: MRC has funded 13 clinical studies, NCATS’ supported studies have led to 14 Investigational New Drug (IND) applications, and the AstraZeneca initiative has resulted in 31 clinical studies. This is amazing productivity and although it is too early to say whether or not any of these studies will lead to new treatments, there are some exciting hints. Thus, Saracatinib, an inhibitor of SRC tyrosine kinase, is a compound originally developed by AstraZeneca for cancer, which is now being investigated as a potential treatment for Alzheimer disease.^5^ It has been shown that saracatinib has activity in a mouse model of Alzheimer disease and that it enters the CNS.^6^ Furthermore, a clinical study has been carried out which confirmed the safety and tolerability of saracatinib in patients with Alzheimer disease.^7^ The drug has been tested in a phase IIa clinical trial to investigate its efficacy in treating Alzheimer disease. This study has been completed and the results are pending. Thus, such initiatives may lead to important new treatments in the future, but perhaps more importantly for the present, they are proving a great way to encourage new and innovative ideas, and the resulting studies are providing invaluable information about human biological systems and diseases.

We believe that there are a number of key learnings from these different initiatives. Gaining access to industry assets has perhaps been more difficult than anticipated, and is often dependent on having an internal champion in the company, close to the decision‐makers. Maintaining partnerships with industry is easier with companies that have dedicated resources for Open Innovation. Provision of interesting compounds is crucial—some compounds are definitely much more popular than others! Provision of expertise, regulatory documentation, and drug supply by Pharma are the ideal scenario; the latter may be unrealistic in some cases, so access to grants like the Wellcome Trust Biomedical Resources Grant provides a good alternative. Once compounds are available, they have resulted in considerable clinical investigational activity, and of a high quality. As the studies are generally carried out within academia, they tend to be small proof‐of‐concept studies, often resulting in trends for effects rather than statistically significant effects. This suggests that there is a need for a mechanism for follow‐up of these small studies, especially if the study is in an area that the originating company is unlikely to pursue. This might involve, for example, a spin‐out company.

Armed with the above learnings and given the significant encouragement obtained from the initiatives which are already running, we look forward to a much better understanding of human biology and disease, a much better understanding of how to translate preclinical findings into positive clinical data, and ultimately the discovery of new and improved medicines.
